# Food intake of early juvenile western Baltic cod (*Gadus morhua*) during settlement transition

**DOI:** 10.1111/jfb.70234

**Published:** 2025-09-24

**Authors:** Anton Höper, Nicole Funk, Felix Mittermayer, Axel Temming, Steffen Funk

**Affiliations:** ^1^ Department of Biology Institute for Marine Ecosystem and Fishery Science, Centre for Earth System Research and Sustainability (CEN), University of Hamburg Hamburg Germany; ^2^ Department of Marine Ecology GEOMAR Helmholtz Centre for Ocean Research Kiel Kiel Germany

**Keywords:** Baltic Sea, *Gadus morhua*, juvenile cod, ontogenetic diet shift, settlement, stomach content

## Abstract

This study examines the gut contents of 203 early juvenile Atlantic cod [17–101 mm ± 18.48 mm standard deviation (SD)] from the Western Baltic Sea (ICES Subdivision 22) collected between 2020 and 2022. According to the observed prey (proportion of pelagic, intermediate and benthic items) in the cod guts, settlement transition from a pelagic to a benthic lifestyle is estimated to take place at 46–87 mm cod total length (TL). Copepod species were the preferred prey item of pelagic feeding juvenile cod, dominated by the genus *Acartia*, which is also the most abundant copepod genus in the area. With increasing cod size, *Centropages* spp. and *Cladocera* species were favoured. Intermediate prey consisted mostly of late bivalve veliger larvae. Although a switch from planktonic to intermediate prey was not observable in every cod individual (probably due to differences in prey availability between years and stations), our results showed that especially at the beginning of the demersal life, all examined cod relied almost exclusively on the Cumacean species *Diastylis rathkei*. Its importance to cod during the settlement transition is in accordance with earlier findings from the same and adjacent areas highlighting it as potential key, but also bottleneck, species for cod recruitment success. Because *D. rathkei* is highly sensitive to low oxygen conditions, and oxygen minimum zones are spreading in the Western Baltic Sea, the decreasing access to *D. rathkei* as prey might be a contributing factor to the low recruitment success of cod in recent years.

## 1 INTRODUCTION

The western Baltic cod (WBC) (*Gadus morhua*, L. 1758) stock, once one of the commercially most important demersal fish species in the area, experienced a drastic decline until its collapse in 2016 (Möllmann et al., 2021; Receveur et al., 2022). Despite efforts to reduce pressure on the stock (‘by‐catch only quota’ for commercial fishers since 2022; ICES, 2021), the stock has not yet shown any signs of recovery.

The constant decline of the WBC stock has been mostly attributed to unsustainable high fishing mortality despite quota reductions [ICES, 2023, increased natural mortality (most likely due to higher predator pressure, e.g., seals and cormorants) and unfavourable temperature conditions; ICES, 2021, Funk et al., 2021] and an extremely variable recruitment (ICES, 2023). Since 2010, recruitment success has been mostly on low levels (Möllmann et al., 2021; Receveur et al., 2022).

The main cause for low recruitment success in the past decade remains unclear so far, but most likely it is driven by multiple stressors acting in concert. Previous studies investigated mainly fecundity and the earliest life stages of cod to find causes for fluctuating recruitment (e.g., Kraus et al., 2000; Lambert & Dutil, 2000; Marshall et al., 1998, 2003; Marteinsdottir & Steinarsson, 1998; Sherwood et al., 2007; Skjæraasen et al., 2006). In contrast, the juvenile stage of cod remains understudied in terms of its potential impact on recruitment. The early juvenile phase of cod, particularly the transition from a pelagic to a demersal lifestyle, along with the associated changes in diet, is considered a critical bottleneck (Campana, 1996; Campana et al., 1989; Hüssy et al., 1997; Tupper & Boutilier, 1995a; Tupper & Boutilier, 1995b; Tupper & Boutilier, 1997). Furthermore, it is assumed that juvenile mortality may have an even greater impact on overall recruitment success than larval and egg mortality (Hüssy et al., 1997; Perry & Neilson, 1988).

Mortality of postlarval and early juvenile stages can be mostly attributed to predation pressure or a combination of negative stressors on the recruiting cohorts (Günther, 2013; Houde, 1987; Tupper & Boutilier, 1995a; Tupper & Boutilier, 1995b). To escape the predation window (as predation in this case is size dependent), rapid growth is a prerequisite to shorten their exposure stage (Houde, 1987; Tupper & Boutilier, 1995a). Because growth is directly dependent on both food quantity and quality (for cod, see e.g., Jobling, 1982; Karlsen et al., 2015), understanding juvenile feeding ecology and food intake mechanisms may help to shed light on its variable recruitment dynamics. A profound knowledge on the food intake of early juvenile cod may furthermore contribute to the identification of suitable indicators, such as the abundance of important prey organisms (e.g., Beaugrand et al., 2003; Nicolas et al., 2014), to enable better predictions of recruitment fluctuations in the future.

To date, knowledge on the feeding ecology of early juvenile WBC is limited. In the past, it has been assumed that observations from adjacent areas, such as the Eastern Baltic Sea with its considerably different hydrography (e.g., Hüssy et al., 1997), are representative of the entire Baltic region. Here the majority of juvenile eastern Baltic cod (EBC) settled at a standard length between 45 and 53 mm. The observed main benthic prey groups during this shift were mysids (*Mysis mixta* L. 1853, *Mysis vulgaris* T. 1828) and amphipods (*Gammarus locusta* L. 1758). However, due to hydrographic differences between the Western and Eastern Baltic Sea (Leppäranta & Myrberg, 2009) and the resulting differences in species occurrences and compositions, this assumption is considered not tenable. Recent studies of Plonus et al. (2021) and Funk (2017) already indicate differences in food composition between areas during the juvenile cod settlement phase pinpointing the importance of regional research efforts in this matter. Altered environmental conditions in this area over the past decades, due to climate change, have forced the juvenile cod to cope with a changing habitat, and thus food regime (MacKenzie et al., 2007).

Here we present a study on the resource utilization of early juvenile WBC. Based on an analysis of stomach contents of juvenile cod from the Belt Sea (ICES Subdivision 22), this study aims at identifying (i) the size and composition of prey species at different juvenile cod feeding phases, (ii) the timing of the ontogenetic feeding shift and (iii) how resource utilization in WBC differs from other Atlantic cod stocks from adjacent areas.

## 2 MATERIALS AND METHODS

### 2.1 Study area

**FIGURE 1:**
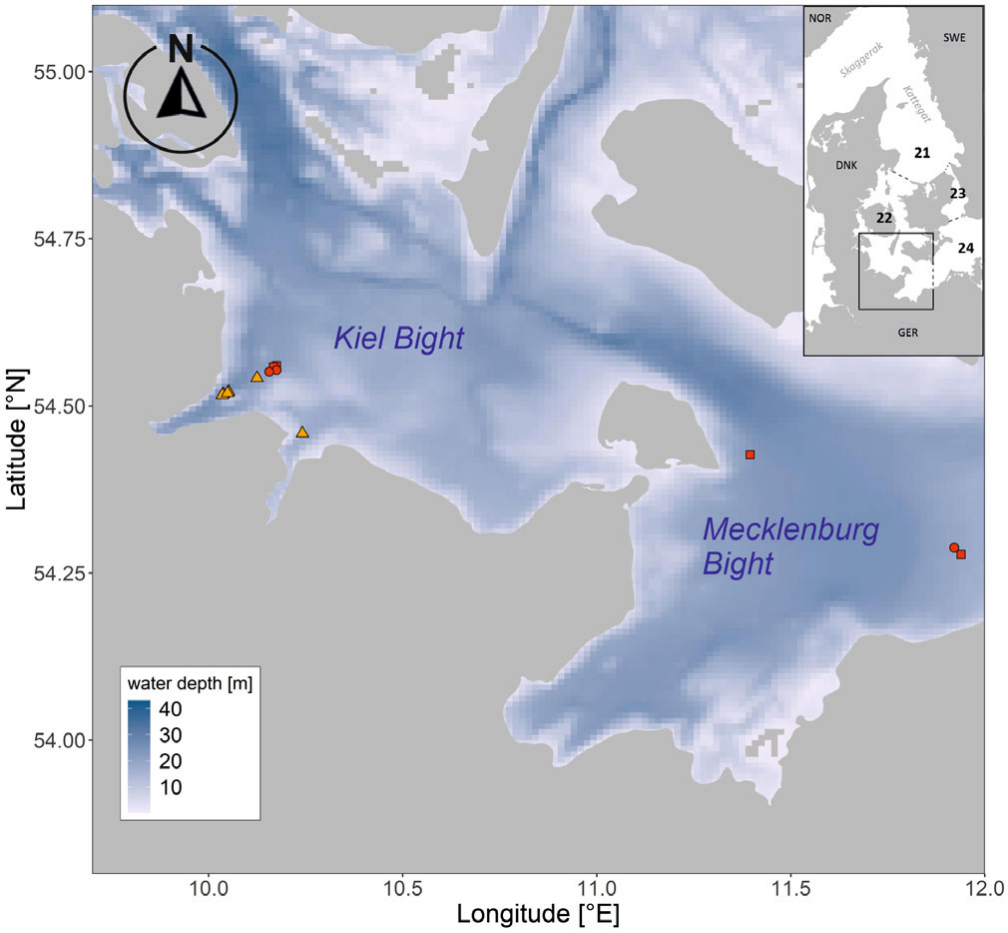
Bathymetric map of the study area displaying sampling positions. Shapes display years (circles = 2020, squares = 2021 and triangles = 2022), and colours display months (orange = June and red = July). Map inlet displays location of the study area (black rectangle) within the Baltic Sea, with dashed lines displaying ICES subdivision borders and numbers indicating corresponding ICES subdivisions (21 = Kattegat, 22 = Belt Sea, 23 = Sound and 24 = Arkona Sea). Long Lat given in decimal degrees.

The sampling of western Baltic juvenile cod was conducted in the Kiel and the Mecklenburg Bight within ICES Subdivision (SD) 22 (Figure 1). The hydrography in this shallow subdivision underlies high fluctuations, which are mainly wind and density driven. Dense high saline water from the north flows into the Baltic Sea, whereas less saline surface water flows from the Central Baltic Sea to the north (Leppäranta & Myrberg, 2009; Snoeijs‐Leijonmalm & Andrén, 2017). The area, therefore, shows a large salinity range and is considered to be brackish (10–25 PSU) (ICES, 2017). The Belt Sea is the main distribution area of the WBC stock, and a mixing with the EBC stock in this region is very unlikely (ICES, 2019).

### 2.2 Cod sampling

A total of 203 samples of early juvenile cod [size range 17−101 mm ± 18.48 mm standard deviation (SD)] were collected between 2020 and 2022. To obtain a reasonable number of samples for our analysis, we decided to collect cod from multiple fishing trips and multiple gears (for more information and summary of gear types and sampling periods, see Supporting Information SI1.1).

Most cod samples were obtained with the research vessel (R.V.) *Alkor* in July 2020 (AL539 and AL540) and 2021 (AL560), as well as in June 2022 (gear‐test day trips) (Figure 1). Additional samples were obtained from research trawling activities on a chartered commercial trawl vessel in June 2022.

For trawling with R.V. *Alkor* in 2020, a pelagic youngfish net was used with 6 mm mesh‐size in the codend. The net was equipped with additional weights to enable trawling close to the bottom. In 2021, in addition to the pelagic youngfish net, the standard bottom trawl (TV3/520; 6‐mm codend mesh‐size) of the Baltic International Trawl Survey was used (see ICES, 2017). On the commercial charter vessel in 2022, a bottom otter trawl with modified codend (24 mm mesh‐size) was used. During the R.V. *Alkor* gear‐test daytrips, an 80‐ft pelagic trawl and a dredge trawl were used with mesh‐sizes in the codend of 10‐ and 11 mm, respectively.

Trawling duration was not standardized between hauls and trips and varied between 8 and 45 min. All catches were sorted on board, and all juvenile cod (i.e., cod with sizes <15 cm) were directly deep frozen at −80°C before being transferred to −20°C for subsequent analysis in the laboratory of the Institute of Marine Ecosystem and Fishery Science at the University of Hamburg.

Cod sampling and removal in the course of the study (including all sources: commercial fishing and scientific surveys) were always carried out in strict compliance with the legal framework of the German Animal Welfare Act (Deutsches Tierschutzgesetz TierSchG).

### 2.3 Processing of the cod samples

In the laboratory, the sampled cod were carefully thawed at room temperature (~20°C). After being thawed, their total length (TL) (accuracy: 1 mm) and full weight (accuracy: 0.1 mg) were measured and noted (KERN EG220‐3NM; Kern & Sohn GmbH). The abdominal cavity was opened, and the stomach was removed using dissecting tools.

Cod showing signs of regurgitation, for example, everted swim bladders or gill rakers attached with remains of food, as well as those showing signs of net feeding (e.g., non‐digested food attached to the gill rakers or the oesophagus), were excluded from the subsequent stomach analysis.

### 2.4 Stomach content analysis

Adherent water was removed from the stomachs, and the stomachs were weighed (accuracy: 0.1 mg, KERN EG220‐3NM; Kern & Sohn GmbH). Subsequently, the stomachs were opened, and the contents were rinsed in a Petri dish using fixing and preservation stock solution consisting of propylene phenoxetol (0.5%), propylene glycol (5%) and deionized H_2_O (94.5%) (Steedman, 1976). Adherent sorting solution was removed from the empty stomachs, and the empty stomachs were weighed. The stomach content weights (SCW) were derived from the differences between full ($W_{\mathrm{FS}}$) and empty ($W_{\mathrm{ES}}$) stomach weights.

For subsequent analysis, the stomach contents were transferred into a Bogorov dish, and pictures of the stomach contents were taken using a stereo microscope equipped with a digital camera (Leica MC190HD). For each stomach sample, a scale has been added to the image for subsequent size measurements of the prey organisms. After imaging, all stomach contents were transferred to a sorting solution in snap‐top vials and archived for future studies. Care was taken to photograph the entire stomach contents; therefore, each subrectangle of the Bogorov dish was recorded and saved as a separate image file.

The subsequent analysis of the image files was performed using Image‐J processing software (ImageJ, version: 1.53 t; Schneider et al., 2012). Prey organisms were identified to species level (certain copepods and cladocerans species), genus level (e.g., *Acartia* spp., *Pseudocalanus* spp. and *Centropages* spp.), order level (e.g., Peracarida), class level (e.g., Bivalvia) or only to phylum level (e.g., Annelida). For all identifiable prey particles (i.e., digestion was progressed only so far that identification was possible), the length was measured using the image‐J software functions ‘straight line’ and ‘segmented line’ in combination with the previously added scale (accuracy: 0.001 mm). Subsequently, the prey weight was recalculated. Depending on the species and its condition, different methods were used: (i) length measurements of prey particles and species‐specific length‐weight relationships for the corresponding species (or for a species or genus considered similar) or (ii) fixed weight estimates for the species and/or species‐specific life stage taken from the literature (for further details, see Supporting Information S2). In case of the occurrence of large prey particles (i.e., TL of several centimetres), these prey particles have been sorted out of the stomachs directly after opening and before rinsing the stomach contents into the Petri dishes. Afterwards, they were weighed and measured individually. In case of most Annelida prey objects, only the setae remained in the stomachs, making length measurements and estimation of prey weights impossible. As a rough approximation of the Annelida weight in the stomach contents, we therefore estimated the approximate percentage of setae in the total stomach contents and calculated its weight relative to the whole SCW.

### 2.5 Relative stomach content compositions

None of the 203 samples obtained showed signs of net feeding or regurgitated stomach contents. Seven stomachs (3.4%) were classified as empty and thus discarded from further analysis. To obtain the relative stomach content compositions, the mean weights per prey type were calculated per 10 mm predator length class. In total, 10 juvenile cod length classes were determined, starting at length class 10–20 mm and ending at length class 101–110 mm. For easier visualization, we further decided to allocate all organisms observed in the stomach contents into eight main prey groups: pelagic Copepoda, Cladocera, other pelagic prey, other intermediate prey, Annelida, *Diastylis rathkei*, Teleostei and other benthic prey (Table 1; for further information on the prey group allocation, see Table SI3.1).

**TABLE 1:** Parameter coefficients (i.e., intercept and slope), explained variance (adjusted R^2^), significance level (*p*‐value; ***: *p* < 0.001), the number of observations adjusted R estimates of linear regressions between cod *Gadus morhua* total length and size of consumed prey organisms (pooled over all prey types and for each prey type separately) and parameter coefficient estimates of corresponding 1% and 99% quantile regressions (i.e., lower and upper).

Prey type	Linear regression				
	Intercept (±SE)	Slope (±SE)	Adjusted R^2^	*p*‐Value	*n*
All prey types	−1.230 (±0.051)	0.035 (±0.001)	0.06	<2.2e−16***	24,879
Pelagic	0.623 (±0.001)	‐	‐	‐	23,412
Intermediate	−2.384 (±0.336)	0.051 (±0.006)	0.16	<2.2e−16***	441
Benthic	−12.183 (±0.675)	0.249 (±0.010)	0.39	<2.2e−16***	1024

	Quantile regression				
	Lower		Upper		
	Intercept (±SE)	Slope (±SE)	Intercept (±SE)	Slope (±SE)	*n*
All prey types	0.0133 (±0.003)	−0.000 (±0.000)	−3.681 (±0.512)	0.160 (±0.009)	24,879
Pelagic	0.0133 (±0.003)	−0.000 (±0.000)	0.880 (±0.030)	0.003 (±0.000)	23,412
Intermediate	0.460 (±0.106)	−0.003 (±0.002)	−7.983 (±0.316)	0.222 (±0.005)	441
Benthic	−0.294 (±0.066)	0.008 (±0.001)	−31.716 (±10.232)	0.843 (±0.184)	1024

Abbreviation: SE, standard error.

### 2.6 Statistical analysis

#### 2.6.1 Predator–prey size relationships

We analysed the dataset for prey size selectivity of cod, wherein we used linear regression models with prey length as response variable and TL of the juvenile cod as explanatory variable. Prey–predator size relationships were calculated for all prey organisms, and for all prey organisms of the class copepoda separately, as copepoda are known as main prey organisms of early juvenile cod from various ecosystems (e.g., Bastrikin et al., 2014; Demain et al., 2011; Hüssy et al., 1997).

We used ordinary least squares (OLS) linear regression to analyse the relationship between prey and cod length (Equation 1):

(1) PL=a+b*L

where PL = prey size, L = TL of the cod and a and b = model parameter estimates.

Following the approach presented in Holt et al. (2019), a quantile regression was used to examine the upper and lower bounds of the prey size distribution (i.e., how minimum and maximum prey sizes scale with TL of the cod), and thus going beyond the pure examination of the average of the Gaussian distribution. The 0.1 and 0.9 quantiles represented the upper and lower bounds, respectively.

#### 2.6.2 Length at settlement transition

We defined the length after Bowman (1981) at settlement transition ($L_{50}$) of cod, as the length at which the probability to be a benthic feeder (i.e., that the majority of the stomach contents in terms of wet weight consisted of benthic prey) was 50% or more. For this purpose, all prey organisms found in the stomachs were classified as pelagic, benthic or intermediate prey respective to the habitat they usually occur in (for further details, see Table SI3.1).

To calculate $L_{50}$, we applied a logistic regression using the relative proportion of benthic prey of the total SCW of each predator as response variable and the TL of the cod as explanatory variable (Equation 2):

(2) $$P=\frac{e^{a+b*L}}{1+e^{a+b*L}}$$

where P = the proportion of benthic food in the stomach content, L = total cod length and a and b = model parameters.

Using the model parameter estimates a and b, we subsequently calculated $L_{50}$ (Equation 3):

(3) $$L_{50}=\frac{\mathrm{Ln}\left(\frac{0.5}{1-0.5}\right)-a}{b}$$

#### 2.6.3 Software used

All calculations and computations were made using the statistical software and programming environment R (R Core Team, 2021). The packages ‘plyr’ (Wickham, 2011), ‘reshape2’ (Wickham, 2007), ‘ggplot2’ (Wickham, 2009), ‘cowplot’ (Wilke, 2017) and ‘quantreg’ (Koenker, 2022) were used.

## 3 RESULTS

### 3.1 Stomach content composition

In total, we analysed 203 stomachs, of which 7 stomachs were classified as empty and excluded from the subsequent analysis (see Table SI4.1).

Observed SCWs ranged between 0.5 and 703.0 mg. Mean SCW per 10‐mm length classes varied between 0.5 and 572.0 mg. Generally, the SCW showed a strong positive trend with increasing cod TL, with a particularly strong increase from the 61–70‐mm length class (see Figure SI4.1).

**FIGURE 2:**
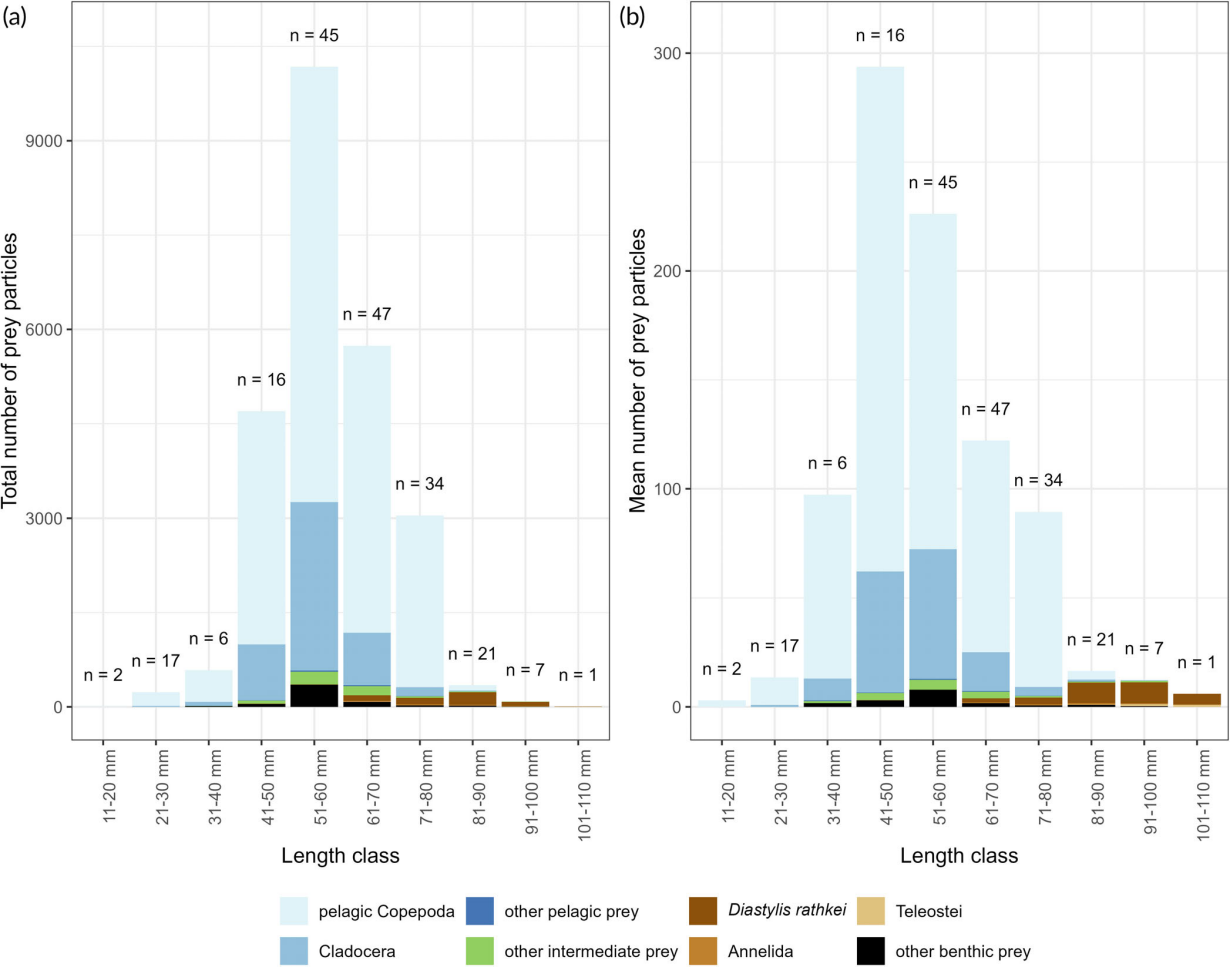
Relative stomach content composition of juvenile cod *Gadus morhua* from SD 22 by number of observed prey particles. (a) Composition of the total number of prey particles (pooled over all stomachs) observed within the 10‐mm length classes of cod per prey group. (b) Mean number of prey particles per prey group and 10‐mm length class of cod. The total number of stomachs per length class is shown above each bar.

Within the 196 full stomachs, we observed 24,981 individual prey particles composed of 32 different prey organisms (see Table SI3.1), which were allocated to eight prey groups: pelagic Copepoda (*N* = 18,597), Cladocera (*N* = 4,635), other pelagic prey (*N* = 48), other intermediate prey (*N* = 442), *D. rathkei* (*N* = 467), Annelida (*N* = 48), Teleostei (*N* = 148) and other benthic prey (*N* = 535) (Figure 2a). Pelagic copepods were the most abundant prey group (by total number of prey particles summed over all cod stomachs) in all cod length classes up to and including 71–80 mm.

Number of prey particles varied greatly among length classes. The mean number of prey particles first show a sharp increase with increasing length, for example, in length class 11–20 mm three (± 1) prey particles were found on average, in length class 41–50 mm 294 (± 156) prey particles. However, with further increasing cod length number of prey particles decrease again, with a sharp drop in mean number of prey particles observable in the length classes 71–80 to 81–90 mm with a mean number of prey particles of 90 (± 170) and 16 (± 13), respectively (Figure 2b).

**FIGURE 3:**
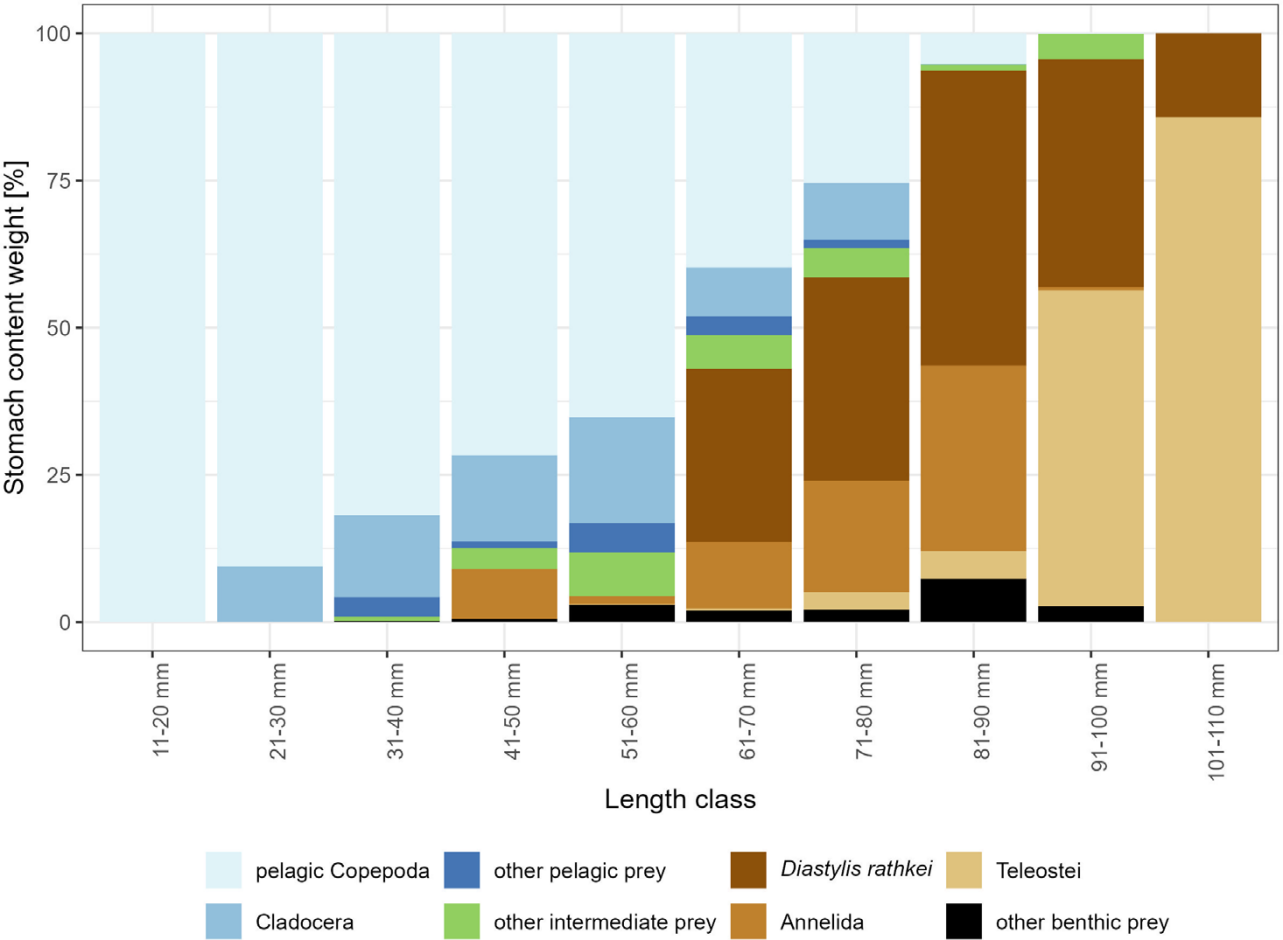
Mean relative stomach content composition (by recalculated prey wet weights) of juvenile cod *Gadus morhua* from SD 22 per 10‐mm length class.

**FIGURE 4:**
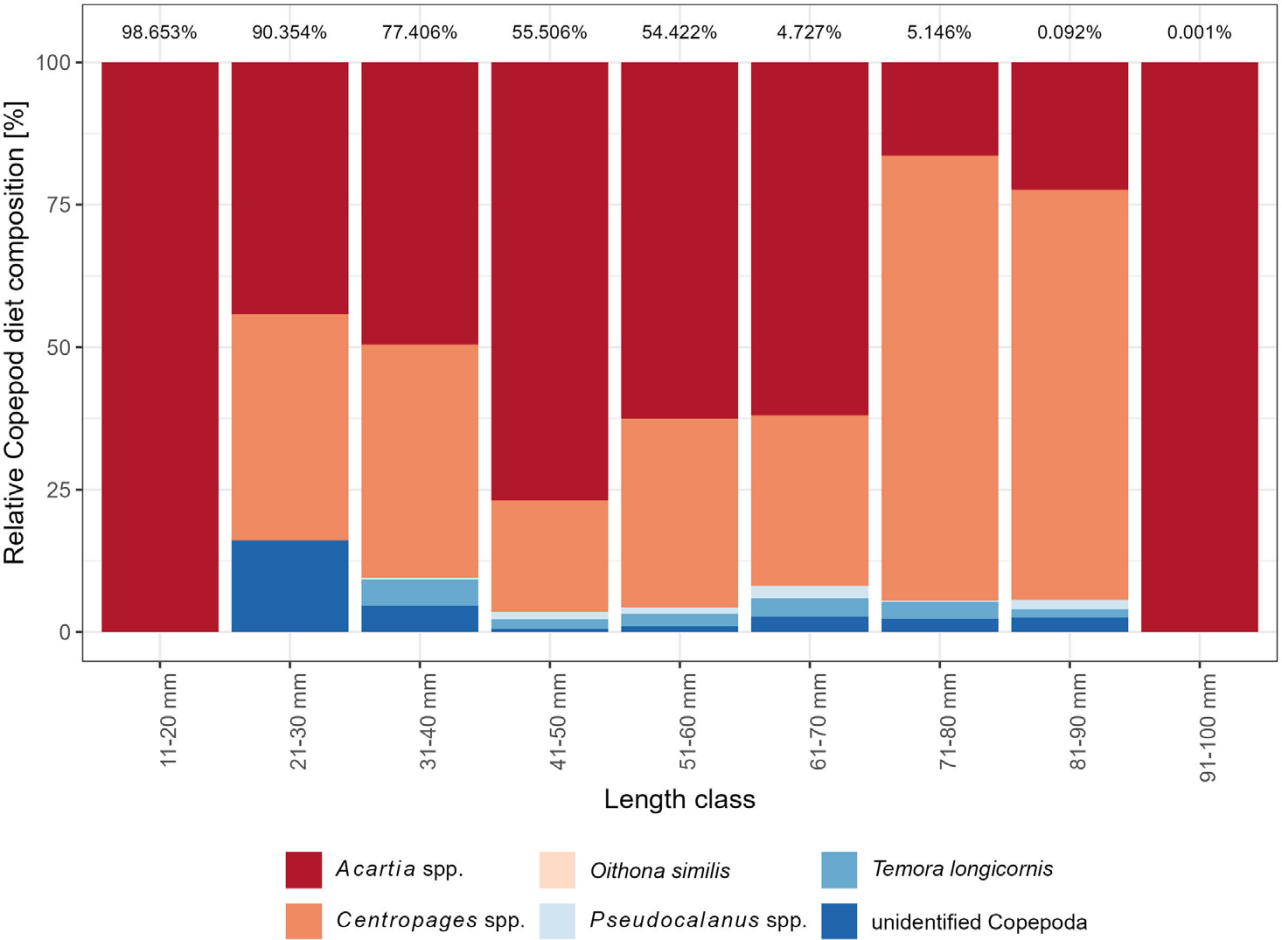
Mean relative Copepoda stomach content composition (by recalculated prey wet weights) of juvenile cod *Gadus morhua* from SD 22 per 10‐mm length class. Cumulated Copepoda weight relative to the whole stomach content per length class is displayed above the bars.

In terms of weight, Copepoda clearly dominated the relative mean stomach content composition for all length classes <61 mm, where they contributed on average to more than half of the total stomach content composition by weight (Figure 3). The group of Copepoda itself was mostly dominated by *Acartia* spp. and *Centropages* spp., whereas *Temora longicorni*s, *Pseudocalanus* spp. and *Oithona similis* occurred relatively rarely and only in lower numbers and weights and thus contributed to only minor shares of the mean Copepoda stomach content composition of early juvenile WBC (Figure 4). With increasing cod length, a clear decreasing trend in the relative contribution of Copepoda prey was visible from 100% (± 0%) in length class 11–20 mm to 65% (± 29%) in length class 51–60 mm, and only 5% (± 22%) in length class 81–90 mm. Furthermore, we observed increasing proportions of Cladocera within the pelagic prey groups with increasing cod length. From the length class 41–50 mm, we observed increasing proportions of benthic prey groups (i.e., mostly Annelida and *D. rathkei*), as well as intermediate prey (Figure 3). From the length class 61–70 mm to the length class 81–90 mm, we observed an increase in the proportion of *D. rathkei*, which dominated the mean relative stomach content composition for the length classes 71–80 and 81–90 mm with mean proportions of 35% (± 42%) and 50% (± 43%), respectively. For the same length classes, we observed increasing proportions of the Annelida prey group with increasing cod length. From the length class 91–80 mm on, we observed a strongly increasing contribution of Teleostei in the stomach contents (mostly small gobies of the genus *Pomatoschistus*) with increasing cod lengths, which clearly dominated the mean relative stomach content composition of cod in the length classes 91–100 mm and 101–110 mm with 54% (± 50%; *n* = 7) and 86% (± 0%; *n* = 1), respectively.

### 3.2 Patterns in predator–prey size relationships

Sizes of observed prey organisms within the cod stomachs ranged between 79 μm and 55.7 mm. Mean prey sizes varied greatly among prey groups [pelagic Copepoda: 0.62 mm (± 0.19 mm), Cladocera: 0.63 mm (± 0.13 mm), other pelagic prey: 0.99 mm (± 0.20 mm), intermediate prey: 0.64 mm (± 1.19 mm), *D. rathkei*: 8.23 mm (± 1.98 mm), Annelida: 11.36 mm (± 13.46 mm), Teleostei: 34.32 mm (± 18.53 mm) and other benthic prey: 0.51 mm (± 0.73 mm)] and also between cod length classes.

**FIGURE 5:**
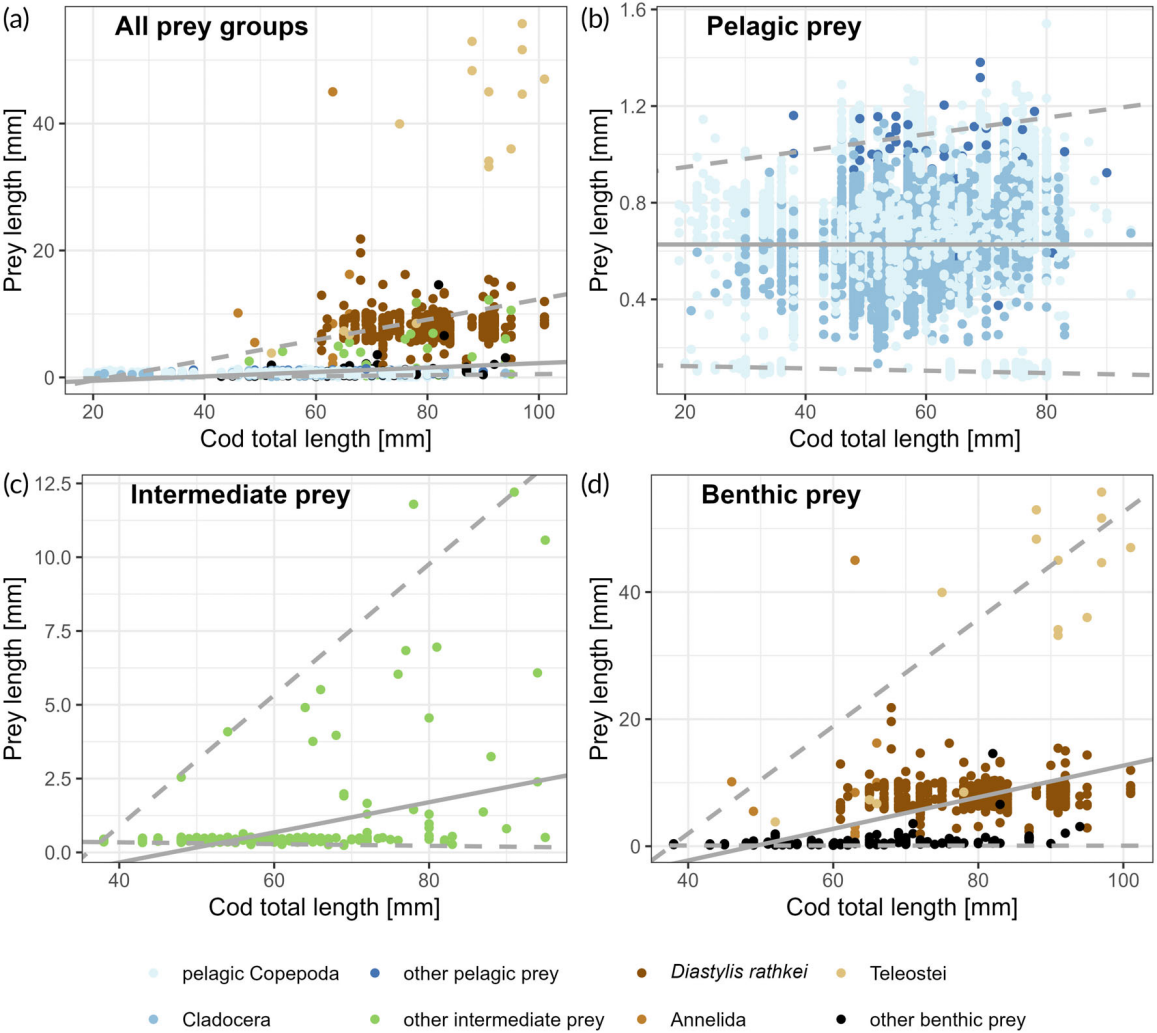
Predator–prey size relationships for juvenile cod *Gadus morhua* from SD 22 and prey organisms (a: all prey groups; b: pelagic prey groups only; c: intermediate prey groups only; and d: benthic prey groups only). Data points depict individual prey particle size observations from the cod stomach samples. Solid grey lines display calculated linear regressions (i.e., the null model and intercept in case of the pelagic prey group only) fitted to predator and prey length. Dashed grey lines display 25% and 75% quantile regression fitted to the data, respectively.

Fitted linear regression between prey sizes of all prey groups and cod TL revealed a significant relationship (*p*‐value <0.05, R^2^ = 0.06). However, the slope of the regression was not very steep (Table 1). The slope of the 99% quantile regression in contrast showed a much steeper increase (Figure 5a), whereas the 1% quantile regression even showed a decreasing relationship between predator and prey size (Figure 5a; Table 1).

For the relationship between pelagic prey length and cod length, the null model (i.e., intercept only) was favoured over the linear regression, revealing no significant increase of pelagic prey length with increasing cod length. The 99%‐quantile regression, however, showed an increasing trend of the 99% quantile of consumed pelagic prey length by increasing cod length. In contrast, the 1% quantile even showed a decreasing tendency of prey sizes in the 1% quantile with increasing cod length, which pointed towards a broadening of the prey sizes with increasing cod length in the direction of both smaller and larger prey (Figure 5b; Table 1).

For the intermediate prey, the fitted linear regression was found to be significant (*p* < 0.05) and thus clearly revealed a positive relationship between cod length and size of consumed prey particles. The 1% and 99% quantile regressions revealed a classical wedge‐shaped pattern, with the 99% quantile showing a much steeper slope than the linear regression, whereas the 1% quantile was slightly negative (Figure 5c; Table 1).

A wedge shape pattern was also observed for the relationship between cod TL and benthic prey sizes. However, the slopes of both the linear regression and 99% quantile regression for benthic prey were several magnitudes higher than those calculated for intermediate prey, and the 1% quantile regression also displayed a positive slope (Figure 5d; Table 1).

### 3.3 Ontogenetic diet shift and length at settlement transition

**FIGURE 6:**
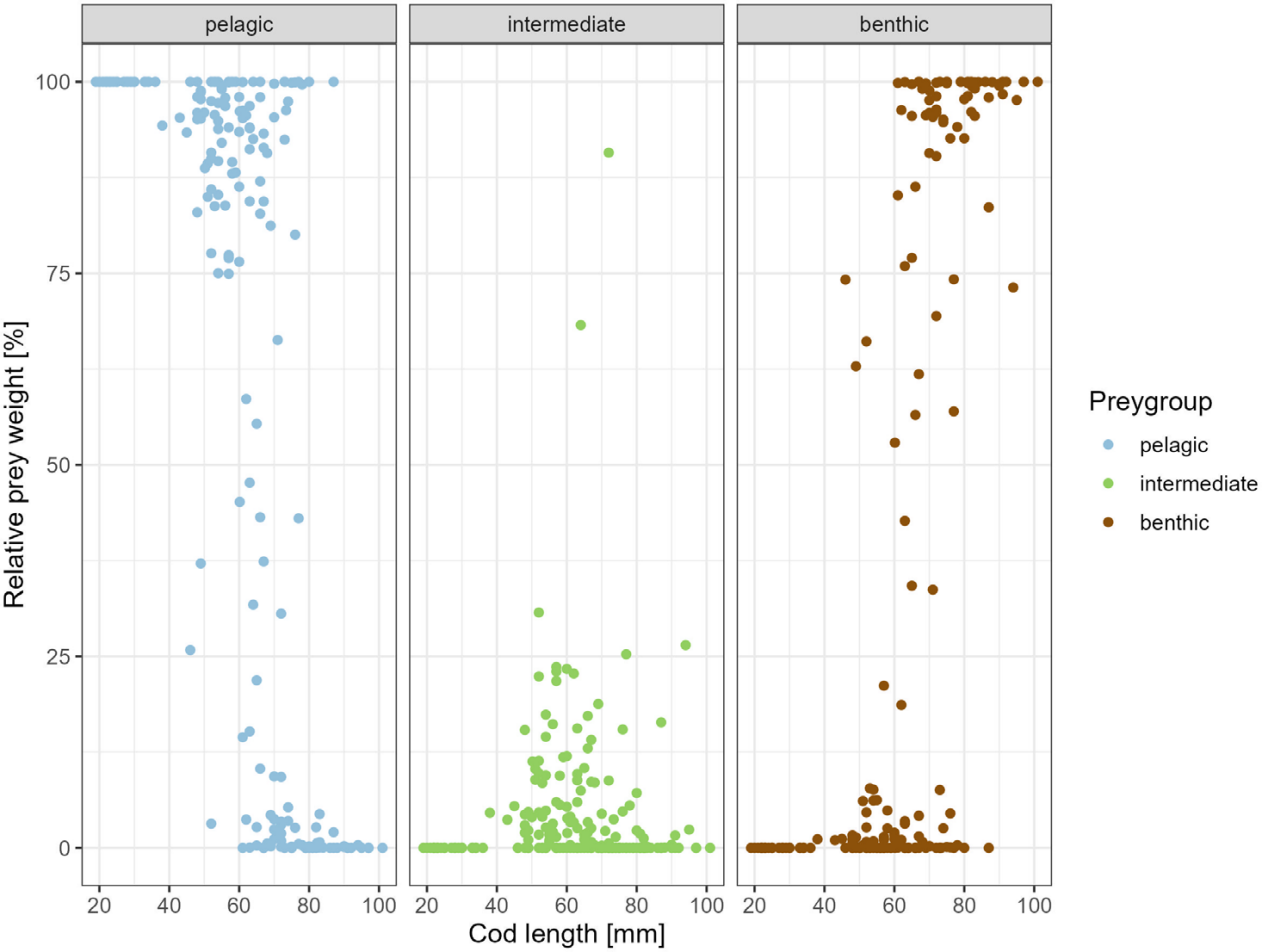
Proportion of prey groups (pelagic, intermediate and benthic) in the stomach contents of juvenile cod *Gadus morhua* by sum of recalculated prey wet weight versus cod total length. Data points represent individual fish.

The observed relative weight proportions of the prey assigned to the pelagic, intermediate and benthic habitats in the stomach content changed markedly with increasing predator length (Figure 6). The length of fish consuming more than 50% pelagic prey ranged from 19 to 87 mm (Figure 6). Stomach contents consisting 100% of pelagic prey were common, particularly in fish <40 mm, but were also observed in larger individuals, including those >80 mm (Figure 6).

Intermediate prey occurred in the stomach contents of fish between 38 and 95 mm (Figure 6). Of the total of 196 stomachs containing prey particles, only 2 showed an intermediate prey proportion of more than 50% with the remaining 194 stomach contents ranging between 0% and 31% (Figure 6).

**FIGURE 7:**
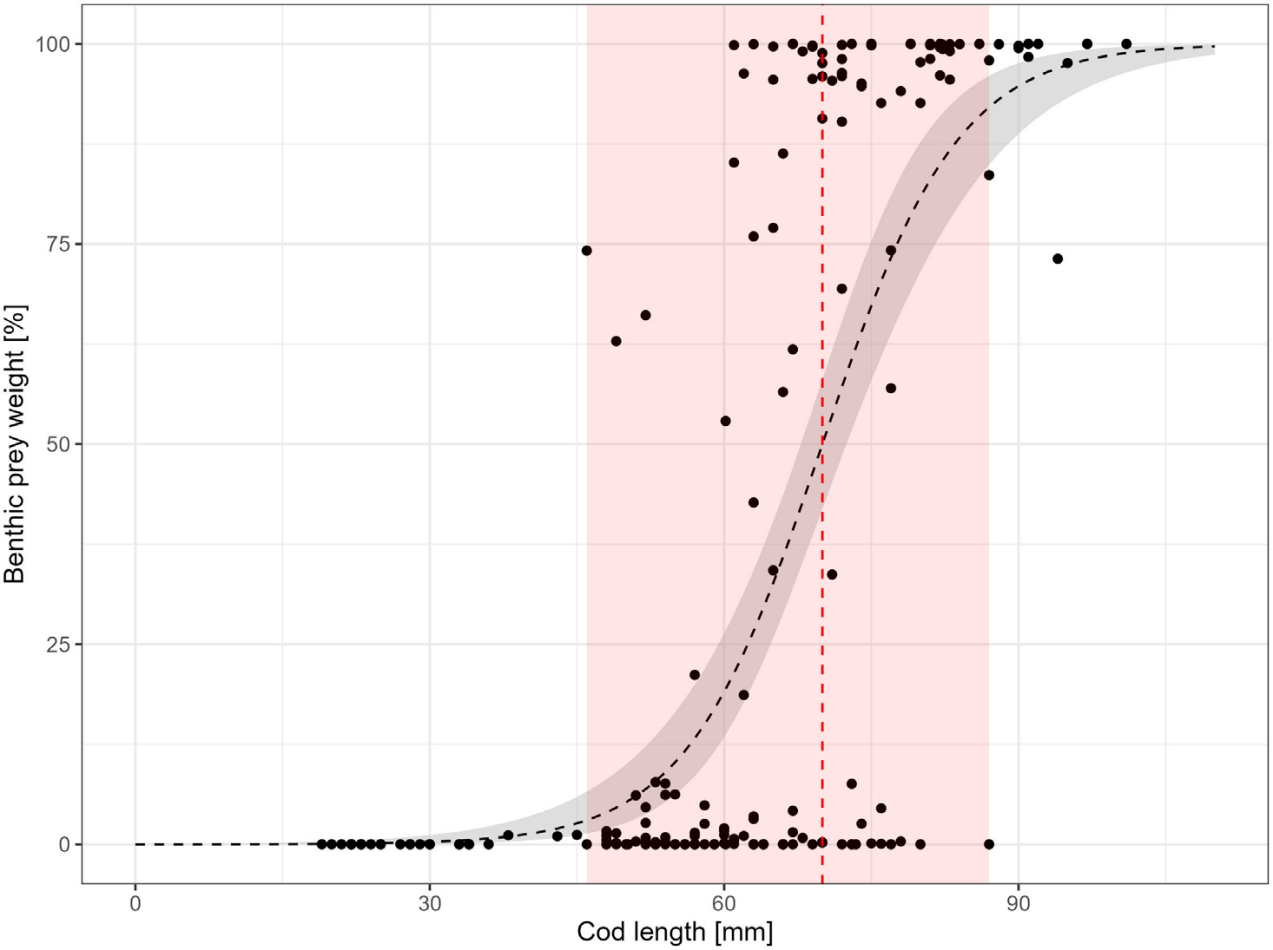
Relation between cod *Gadus morhua* total length and proportion of benthic prey. Data points represent individual fish. The dashed line displays a binomial logistic regression. The grey shaded area depicts confidence intervals. The dashed red line indicates the calculated length at settlement transition *L*_50_ (=70.01 mm). The shaded red area displays the hypothetical length range of settlement transition starting from the smallest individual (46 mm), where benthic prey amount ≥50% of the total stomach content weight, and the largest individual (87 mm), where <50% of the total stomach content weight consisted of benthic prey.

Benthic prey was observed to contribute more than 50% of the SCW in individuals of fish lengths between 46 (74%) and 101 mm (100%), with the smallest fish consuming exclusively benthic prey measuring 67 mm (Figures 6 and 7). The TL of the largest fish consuming less than 100% benthic prey was observed to be 95 mm (Figures 6 and 7).

The whole phase of settlement transition of the juvenile cod examined can be assumed to take place between 46 and 87 mm TL, as these lengths comprised both the smallest fish consuming ≥50% benthic prey and the largest fish consuming ≤50% benthic prey (Figure 7). The fitted logistic regression (a=−10.12 and b=0.14) revealed an *L*_50_ of 70.01 mm (Figure 7).

## 4 DISCUSSION

In this study, we investigated the food resource utilization of early juvenile cod from the Western Baltic Sea to identify critical prey species. Furthermore, the transition timing from a pelagic to a benthic lifestyle and potentially stock‐ or spatial‐specific patterns were analysed.

### 4.1 Patterns in early juvenile western Baltic cod diets

The prey items found in juvenile cod stomach contents were distributed into pelagic, intermediate and benthic, of which only intermediate and benthic prey showed a significant trend of larger predators consuming prey of larger size. Each prey category is discussed in greater detail below.

### 4.2 Pelagic prey

Similar to studies from the North Sea and Eastern Baltic Sea, we found pelagic copepod species to be the main prey of pelagic feeding cod in the Western Baltic Sea (Bastrikin et al., 2014; Hüssy et al., 1997). Although in other areas cod have been observed utilizing mostly relatively large copepod species of genus *Pseudocalanus* or *Calanus* (Baltic Sea: Hüssy et al., 1997; Norwegian Trench: Shaw et al., 2006; North Atlantic: Jacobsen et al., 2020; North Sea: Bastrikin et al., 2014), early cod juveniles in the Western Baltic Sea seem to rely on smaller copepod genera *Acartia*, which is the most abundant genus in the area (Dutz et al., 2022). This would be in line with previous studies suggesting that juvenile cod generally are non‐selective and feed on the most abundant prey items available (Daan, 1973; Keats & Steele, 1992; Mattson, 1990). However, there may be evidence in our data that at least size‐selective feeding behaviour does occur to some extent. For the years 2020 and 2022, for example, the proportion of *Centropages* spp. increases with increasing cod length (see Figure SI5.1). However, this trend cannot be seen in 2021. As there is unfortunately no good spatial and temporal coverage of data on copepod abundancies for the investigated area, it can be speculated only whether the trend is due to abundance shifts in the copepod community or whether, with similarly high abundances of *Acartia* spp. and the larger *Centropages* spp. in recent years (Dutz et al., 2022), *Centropages* spp. were indeed favoured by the cod with increasing size.

Besides increasing proportions of *Centropages* spp., we observed increasing proportions of the comparably larger *Cladocera* species such as *Podon* spp. and *Evadne* spp.

However, the observed proportions of cladocerans in the stomach contents were significantly smaller than those of the pelagic copepods. This is in contrast to observations by Hüssy et al. (1997), who found larger proportions of cladocerans in the stomach contents and, therefore, assigned a higher importance to them, whereas they are not reported to be of greater importance for juvenile cod from the North Sea and North Atlantic region (Bastrikin et al., 2014; Jacobsen et al., 2020). Cladocerans show relatively low abundances in the Kiel Bight and Mecklenburg Bight (Dutz et al., 2022). Recent biological assessments of the Baltic Sea indicate a low abundance anomaly of zooplankton in the past decade (Dutz et al., 2022; Kremp et al., 2024). Therefore, we assume that the cod investigated in this study encountered these prey items more rarely, leading to lower consumption rates reflected in the gut contents.

Furthermore, our linear regression analysis did not reveal a significant positive linear relationship between predator and pelagic prey size. Instead, it showed a rather constant utilization of plankton prey of the same size range. A closer look at the fitted quantile regressions, however, points towards a prey size spectrum broadening at the upper and lower ends, that is, towards the utilization of larger and smaller plankton prey sizes with increasing cod length. For example, some larger cod had higher amounts of small copepods consumed as the average in their length class. Following Houde (1997), this could be their strategy to cover increasing energy requirements by maximizing prey encounter rates when large plankton prey is scarce.

### 4.3 Intermediate prey

For the intermediate prey, which consisted mostly of late bivalve veliger larvae, we observed highest consumption proportions at cod sizes between 52 and 72 mm where the settlement transition is taking place (individual proportions >30%). This stands in line with the results of Hüssy et al. (1997), who found that EBC utilizes mysids, classified here as intermediate prey, mostly at lengths where the settlement transition occurred. However, Mysidacea tend to be more abundant in the Central Baltic Sea compared to the western parts of the Baltic Sea (Zettler, 2001), and that abundances fluctuate strongly with season (Arntz, 1971). This could explain why smaller proportions have been found in our samples compared to Hüssy et al. (1997).

During settlement, cod starts to orient itself towards the bottom during its food intake and probably tests different alternative prey types to maximize encounter rates and to meet the increasing energy demands to maintain continuous growth. In contrast to the pelagic prey, our logistic regression analysis revealed a statistic significance for the utilization of larger intermediate prey with increasing prey length, which emphasizes the assumption that the shift from pelagic to intermediate prey results from an ongoing size‐selective feeding behaviour. Thus, intermediate prey plays an important role as a linkage between the pelagic and benthic habitat usage during the transition phase.

### 4.4 Benthic prey

Although a switch from planktonic to intermediate prey does not appear in every cod individual in the Belt Sea (probably due to differences in prey availability between years and stations), the transition to a benthic diet has been observed in all individuals >87 mm. Our results showed that, particularly at the beginning of the demersal life, cod seem to rely almost exclusively on the Cumacean species *D. rathkei*, clearly highlighting it as a key prey species for cod in the area. The importance of *D. rathkei* to cod during settlement transition is in accordance with earlier findings by Funk (2017) in the same area and Hüssy et al. (1997) for the Eastern Baltic Sea. However, in the Eastern Baltic Sea, *D. rathkei* was only one of several benthic invertebrates, with amphipods mostly dominating the overall stomach content composition of the early settled cod. This may be attributed to the difference in the composition of the benthic community between the Eastern and Western Baltic Sea. In the Western Baltic Sea, *D. rathkei* tends to dominate the hyperbenthic community (Arntz, 1971; Zettler, 2001). Rumohr et al. (1996) and Arntz (1971) furthermore observed this species to be a typical taxon in deep regions (10–29 m) of the Western Baltic Sea.

Similar to our observations for intermediate prey, our statistical analyses on predator–prey size selectivity for benthic prey revealed a clear positive relationship, which was mostly driven by the occurrence of teleosts, in particular gobies of the genus *Pomatoschistus*, in the stomach contents of cod >80 mm. The wedge‐shaped patterns in the predator–prey size relation, highlighted by the 1% and 99% quantile regressions (Figure 5c,d), are typical in cod (e.g., Holt et al., 2019) and are also observed in many other predatory fish species (e.g., Pinnegar et al., 2003). Pinnegar et al. (2003) suggest that the diet choice of a predator is the result of balancing costs and benefits, considering the quality of the prey and the energy invested for search, capture and handling. The frequent occurrence of *D. rathkei* in the cod stomachs of WBC is probably a result of both, comparable high densities and relatively low energy expenditure to capture prey.

This is also highlighted by previous studies in which *D. rathkei* is described as an important food item in muddy bottom areas for demersal fish species, including immature cod (Arntz, 1980; Arntz & Finger, 1981; Rachor et al., 1982).

Because *D. rathkei* seems to play a key role during and after settlement of WBC, we argue that its density and size structure could play decisive roles in the timing of WBC settling and, thus, eventually for the overall recruitment success of the WBC stock.

Abundances of *D. rathkei* are mostly controlled by physical factors such as oxygen content in bottom water layers (Rachor et al., 1982). Because the occurrence of oxygen minimum zones (OMZ) in the Western Baltic Sea has increased in frequency, duration and intensity in the past years (Piehl et al., 2023), it can be assumed that this has considerably limited the occurrence of *D. rathkei* and other benthic prey, with high oxygen demands serving as food supply for early cod settlers (Haselmair et al., 2010). Not to mention the direct negative effects of hypoxia on juvenile cod themselves (Chabot & Claireaux, 2008). Oxygen levels in our sampling area, for example, stayed below 4 mg l^−1^ throughout the summer and into the autumn (Hansen & Rytter, 2020; Hansen & Rytter, 2021; Hansen & Rytter, 2022), increasingly occurring in the deeper areas >20 m of the Western Baltic Sea in the past decades.

### 4.5 Regional differences in settlement transition timing

Settlement transition from pelagic to benthic feeding in juvenile cod has been described for different ecoregions earlier (Bastrikin et al., 2014; Bowman, 1979, 1981; Hüssy et al., 1997; Jacobsen et al., 2020; Lomond, 1998; Tupper & Boutilier, 1995a; Tupper & Boutilier, 1995b; Tupper & Boutilier, 1997). However, to the knowledge of the authors, this is the first comprehensive study describing the ontogenetic feeding shift in juvenile WBC. Table 2 summarizes the reported cod lengths at settlement for the adjacent regions of the Baltic Sea and the North Sea. The only data for the adjacent Eastern Baltic Sea region come from Hüssy et al. (1997), which cover only the eastern part. In this region, settlement takes place at cod size between 46 and 80 mm (Hüssy et al., 1997), which is very similar to our results. However, Hüssy et al. (1997) reported that most of the fish finished settling at a TL of >58 mm (i.e., 53 mm standard length), and thus, at considerable smaller TL than observed for WBC (70 mm). Thus, our findings indicate that WBC seems to settle considerably later in individual life history compared to its EBC conspecifics. These findings are supported by otolith microstructure analyses of EBC and WBC, which showed that EBC settlement occurs at an age of ~70 days (Hinrichsen et al., 2009; Hüssy et al., 2003), whereas WBC settlement occurs at an individual age between 90 and 120 days (Plonus et al., 2021). Furthermore, our data suggest a rather gradual change in prey item choice over the course of ontogeny for WBC, whereas EBC shows a rather sharp feeding shift from pelagic to benthic prey at a certain length (Hüssy et al., 1997).

**TABLE 2:** Reported size ranges for the settlement transition of Atlantic cod from the North Sea and Eastern Baltic Sea.

Cod length (mm)	Region	Source
46–87 mm	Western Baltic Sea	This study
46–80 mm^a^ 42–73 mm SL	Eastern Baltic Sea	Hüssy et al. (1997)
49 mm	North Sea	Bastrikin et al. (2014)
50–60 mm	North Sea	Robb and Hislop (1980)

*Note*: All cod lengths are given in total length. Otherwise indicated with SL for standard length.

^a^ Conversion from SL to TL. See Supporting Information SI6.1.

A gradual feeding shift in juvenile cod was also reported by Bastrikin et al. (2014) for the North Sea, as well as by Bowman (1979; Georges Bank) and Pálsson (1980; Iceland) in other ecoregions. Bastrikin et al. (2014) observed first benthic prey in cod sizes ranging from 30 to 135 mm. Because in this study only cod TL < 101 mm were investigated, an occurrence of single pelagic particles in WBC stomach contents of TL > 101 mm cannot be excluded but is considered rather uncommon or fed ‘by chance’. The *L*_50_ calculated by Bastrikin et al. (2014) for North Sea cod was considerably lower at 49 mm compared to the *L*_50_ calculated in the present study for WBC at 70 mm, suggesting that most WBC settle at a larger size than their North Sea conspecifics.

The reasons for both, the considerable larger settlement transition in WBC and the more gradual transition in the feeding shift, compared to EBC can be hypothesised only so far. It is known that planktonic organisms in the Baltic Sea are often associated with thermo‐ and/or haloclines (Schulz & Hirche, 2004; personal observation). In the Belt Sea, thermo‐ and/or haloclines are often found in layers near the bottom in late spring and summer in depths between 15 and 20 m (Gibson et al., 2002; Gräwe et al., 2013; personal observations). Thus, the spatial separation of benthic and pelagic prey may not be as distinct in these shallow areas as it is in the deeper settlement grounds of the EBC. Eventually, this indistinct separation might result in WBC encountering and ingesting both planktonic and benthic prey while feeding, in contrast to the EBC when transitioning to a bottom‐orientated lifestyle. Another possible explanation could be a lack of important benthic prey organisms such as *D. rathkei* in adequate sizes or densities at the settlement grounds.

High diet shares of *D. rathkei* have been observed in both adult and juvenile cod in the past (Arntz, 1974) and in recent studies from the Belt Sea area (Funk, 2017; Funk et al., 2021), where they have been associated with mass occurrences of *D. rathkei*.

However, especially for the sampling years, a limited supply of *D. rathkei* can be assumed, which may be one of the main factors for the relatively large settling size observed in this study compared to other cod stocks from adjacent areas. The sampling years were characterized by strong hypoxic conditions (Hansen & Rytter, 2020; Hansen & Rytter, 2021; Hansen & Rytter, 2022), which most likely resulted in a limited occurrence of *D. rathkei* due to their sensitivity to oxygen deficiency (Gibson et al., 2002). Hypoxia in the Western Baltic is known to occur mostly during summer (i.e., from May to September), and mainly in deeper areas (i.e., >15 m), and, thus, both during settling time of WBC and at depths where WBC settling takes place. This hypothesis is supported by lower *L*_50_ and an abrupter transition to the demersal lifestyle of WBC reported by Funk (2017) and Plonus et al. (2021) sampled in years with low summer hypoxia (Hansen & Rytter, 2016), which coincided with observations of *D. rathkei* mass occurrences in stomach contents of both adult and juvenile cod (Funk, 2017; Funk et al., 2021).

### 4.6 Data scarcity

Although we found strong indications that successful settlement transition in WBC might be driven by the availability of *D. rathkei*, we are missing important prey datasets on long‐term trends to underpin this hypothesis. Existent datasets (Dutz et al., 2022; Kremp et al., 2024) mainly focus on general trends in planktonic abundances and dynamics and, thus, do not include detailed information about specific prey organisms relevant for juvenile cod.

However, a spatially and seasonally resolved sampling would be a prerequisite to better understand the mechanisms driving juvenile settlement success, general food web ecology of cod and may present an opportunity to resolve the main drivers for cod recruitment dynamics. Ideally, combined monitoring of juvenile cod and their prey would occur at high spatial and temporal resolution. Detailed knowledge about fluctuations in important prey types of juvenile cod may even act as ‘indicator species’ for recruitment success of the WBC stock.

Furthermore, most investigations on settlement transition of cod were conducted >10 years ago and thus may not reflect the current situation in the ecosystems anymore. This may be especially true for the rapidly changing Baltic Sea, which experienced drastic abiotic and biotic changes in the past decades, reaching from more frequent heatwaves (Pinto et al., 2024), expanding oxygen minimum zones (Carstensen et al., 2014) to regime shifts in species composition (Receveur et al., 2022), with even more severe scenarios predicted for the future (Reusch et al., 2018).

### 4.7 Conclusion

The present results suggest that the settlement transition in WBC, as proven in other ecoregions, is an important early life phase and not negligible in the course of understanding annual recruitment dynamics. Certain prey organisms, such as *D. rathkei*, play a key role after the habitat transition and hold the potential of an ‘indicator’ for recruitment success. However, more spatially and temporally overlapping data on both predator and prey are needed to validate this prospect. Even more so, because the Western Baltic Sea ecosystem has undergone and still undergoes rapid changes, it is crucial to understand the relationship between juvenile cod and prey, as prey may represent a bottleneck that determines year class strength.

## AUTHOR CONTRIBUTIONS

Anton Höper: writing – original draft, visualization, methodology, investigation, formal analysis, data curation, conceptualization. Nicole Funk: writing – review and editing, validation, investigation, data curation, project administration, conceptualization. Felix Mittermayer: writing – review and editing, sampling. Axel Temming: writing – review and editing, validation, supervision, conceptualization. Steffen Funk: writing – original draft, visualization, methodology, investigation, formal analysis, data curation, project administration, resources, supervision, conceptualization. All authors have read and agreed to the published version of the manuscript.

## FUNDING INFORMATION

This study received financial support by the Federal Ministry of Education and Research (BMBF) of Germany in the framework of the project *SpaCeParti* (Coastal Fishery, Biodiversity, Spatial use and climate change: a participative approach to navigate the Western Baltic Sea into a sustainable future; grant no. 03F0914). Further funding was received for Nicole Funk from the BMBF‐funded project *balt_ADAPT* (Adaptation of the Western Baltic coastal fishery to climate change; grant no. 03F0863D) and from the European Union's Horizon Europe research and innovation programme MarinePlan (Improved transdisciplinary science for effective ecosystem‐based maritime spatial planning and conservation in European Seas; grant no. 101059407). *The funders had no role in study design, data collection and analysis, decision to publish or preparation of the manuscript*.

## CONFLICT OF INTEREST STATEMENT

The authors declare that they have no known competing financial interests or personal relationships that could have appeared to influence the work reported in this paper.

## Supporting information


**Data S1.** Supporting information.

## Data Availability

The data that support the findings of this study are available from the corresponding author upon reasonable request.
