# Supplementary material for: Food intake of early juvenile western Baltic cod (Gadus morhua) during settlement transition
Source: J Fish Biol. 2025 Sep 24;108(1):258–71. doi: 10.1111/jfb.70234 (PMC13033972; doi:10.1111/jfb.70234)
Supplement: Supplementary file 1 — Data S1. Supporting information. [file JFB-108-258-s001.docx]

**SUPPORTING INFORMATION**

**SI1. Details on the origin of stomach samples**

**Supporting Information Table SI1.1.** List of cruises by the University of Hamburg and GEOMAR from 2020 to 2022 used for the collection of early juvenile stomach samplings including sampling year and month, vessel information (Alkor = RV Alkor, chartered commercial fishing vessel = SK14), cruise acronym, gear information, number of hauls per gear, number of stomach samples, and length range of collected early juvenile cod samples (i.e., range of total lengths).

| Year | Month | Vessel | Cruise acronym | Gear | No. of hauls | No. of samples | length range |
| --- | --- | --- | --- | --- | --- | --- | --- |
| 2020 | July | Alkor | AL539 | pelagic young fish trawl | 2 | 87 | 49-101 mm |
|  | July | Alkor | AL540 | pelagic young fish trawl | 1 | 2 | 72-81 mm |
| 2021 | July | Alkor | AL560 | bottom trawl (TV3/520) | 5 | 59 | 38-87 mm |
|  | July | Alkor | AL560 | pelagic young fish trawl | 1 | 2 | 46-66 mm |
| 2022 | June | SK14 | daytrip | otter bottom trawl | 1 | 13 | 46-69 mm |
|  | June | SK14 | daytrip | dredge trawl | 1 | 9 | 64-80 mm |
|  | June | Alkor | daytrip | pelagic 80feet youngfish trawl | 1 | 24 | 19-36 mm |
|  |  |  |  |  |  |  |  |

**SI2. Recalculation of prey wet weights**

**Supporting Information Table SI1.2.** List of prey types with the respective type of size measurement, the fixed weight or length-weight relationship and the describing reference.

| Prey | Size measurement | Fixed weight [µg] / Length-Weight relationship | Weight reference |
| --- | --- | --- | --- |
| *Acartia* spp. | Length of cephalothorax | 20 µg | Hernroth (1985) |
| *Centropages* spp. |  | 42.5 µg |  |
| *Temora longicornis* |  | 42.5 µg |  |
| *Pseudocalanus* spp. |  | 42.5 µg |  |
| *Oithona similis* |  | 9 µg |  |
| Copepoda |  | 23.5 µg |  |
| Nauplii |  | 2.25 µg |  |
| *Podon* spp. | Longest part of the body according to Hernroth (1985) | < 0,3 mm = 2 μg  0,3 mm – 0,4 mm = 6 μg  0,4 mm – 0,5 mm = 13 μg  0,5 mm – 0,6 mm = 25 μg  0,6 mm – 0,7 mm = 40 μg  0,7 mm – 0,9 mm = 70 μg  0,9 mm – 1,1 mm = 140 μg  > 1,1 mm = 200 μg |  |
| *Bosmina* spp. |  | < 0,3 mm = 2,5 μg  0,3 mm – 0,4 mm = 7 μg  0,4 mm – 0,5 mm = 15 μg  0,5 mm – 0,6 mm = 25 μg  0,6 mm – 0,7 mm = 45 μg  0,7 mm – 0,9 mm = 80 μg |  |
| *Bosmina coregoni* |  |  |  |
| *Evadne nordmannii* |  | < 0,3 mm = 2 μg  0,3 mm – 0,4 mm = 6 μg  0,4 mm – 0,5 mm = 10 μg  0,5 mm – 0,6 mm = 20 μg  0,6 mm – 0,7 mm = 30 μg  0,7 mm – 0,9 mm = 50 μg  0,9 mm – 1,1 mm = 90 μg  > 1,1 mm = 140 μg |  |
| Calanoid Copepoda egg | Diameter | $Volume= \frac{4}{3}* \pi*r^{3}$ | Assumption that the eggs encountered are pelagic eggs. The density was calculated with the CTD-profile of the according station (highest encountered density at the bottom). |
| Fish egg |  |  |  |
| Cypris larvae | Width of the shells | $Log10\left( W\left[ mg \right] \right)=2.326*Log10\left( L\left[ mm \right] \right)-0.354$ | Approximation based on the equation of Bivalvia spp.; Rumohr et al. (1987) |
| Zoea larvae | Carapace length | Weight determination with Zoea stage (i.e., fixed values per stage) | Dawirs 1980; Mohamedeen and Hartnoll 1989 |
| Bivalvia | Width of the shells | $Log10\left( W\left[ mg \right] \right)=2.326*Log10\left( L\left[ mm \right] \right)-0.354$ | Rumohr et al. (1987) |
| Mysida | Total length | $W[mg]=5.926*{DW\left[ mg \right]}^{0.83}$  $DW\left[ mg \right]=0.00126* {L[mm]}^{3.07}$ | Hansson et al. (1990); Rudstam (1989) |
| Megalopa larvae | Carapace length | Weight determination with Megalopa stage (i.e., fixed values per stage) | Dawirs 1980; Mohamedeen and Hartnoll 1989 |
| *Diastylis rathkei* | Thoraxic length | $W\left[ mg \right]=0.102*{L[mm]}^{2.902}$ | Rachor et al. (1982) |
| Polychaeta/Annelida | Total length  (if organims was measurable) | $Log10\left( W\left[ mg \right] \right)=2.672*Log10\left( L\left[ mm \right] \right)-1.376$ | Rumohr et al. (1987) |
| Polychaeta/Annelida | In case that only Setae have been left: Approximation of the percentage of occurring setae | Percentage of the stomach content weight |  |
| Teleostei | Total length | $Total length\left[ mm \right]= -23.36+56.94*Otolith width[mm]$  $Weight\left[ g \right]=0.1677*{Otolith width\left[ mm \right]}^{5.369}$ | Härkönen (1986) |
| *Pomatoschistus minutus* | Total length / Otolith width |  |  |
| Halacaroidea | Length measurements similar to *Bosmina* spp. | Fixed weights of *Bosmina* spp. used, as the morphology is similar | Approximation based on Hernroth (1985) |
| Harpacticoid copepod | Length of cephalothorax | Fixed weight of 8 µg | Approximation based on Hernroth (1985) |
| Gastropoda | Height oft the shell | $Log10\left( W\left[ mg \right] \right)=2.308*Log10\left( L\left[ mm \right] \right)-0.543$ | Rumohr et al. (1987) |
| *Hydrobia* spp. |  |  |  |
| Foraminifera | Diameter | Comparison with genus *Globorotalia* and *Globoquadrina* | Takahashi et al. (1984) |
| Amphipoda | Total length | $W\left[ mg \right]=0.0327*{L[mm]}^{2.897}$ | Costa and Costa (1999) |
| Gammaridae |  |  |  |
| Ostracoda | Width of the shells | $Log10\left( W\left[ mg \right] \right)=2.326*Log10\left( L\left[ mm \right] \right)-0.354$ | Approximation based on the equation of Bivalvia spp.; Rumohr et al. (1987) |
| *Crangon crangon* | Total length | $Log10\left( W\left[ g \right] \right)=-4.420+2.647*Log10(L\left[ mm \right])$ | Robinson et al. (2010) |

**SI3. Allocation of prey organisms to habitat types**

We classified all observed prey organisms in the stomachs as pelagic, benthic, or intermediate depending on the habitat of the prey item at the time samples were collected, based on information from the literature.

In case of Bivalvia we exclusively observed late Veliger larvae in the stomachs of cod. Veliger larvae of Bivalves show a transition from pelagic to benthic habitats. However, when this settling transition exactly occurs is size-dependent and variable between species (see e.g., Jansson et al. 2015, Martel et al. 2014). Since, no further species identification was possible using visual determination only, we could not further specify whether these Veliger larvae have been already settled or not and hence we decided to allocate all Bivalves observed to the intermediate prey type (Supporting Information Table SI3.1).

**Supporting Information Table SI3.1.** List of prey organisms identified in the stomachs of juvenile cod *Gadus morhua* samples (N = 196) from SD 22 between 2020 and 2022. Prey group lists the groups in which prey organisms were grouped for relative stomach content composition calculations. Allocated prey type displays grouping of observed prey organisms in the 3 major types pelagic, benthic and intermediate respective to the main habitat of occurrence of the prey.

| Prey | Prey Group | Prey type |
| --- | --- | --- |
| *Acartia* spp. | pelagic Copepod | pelagic |
| *Centropages* spp. |  |  |
| *Temora longicornis* |  |  |
| *Pseudocalanus* spp. |  |  |
| *Oithona similis* |  |  |
| Copepoda |  |  |
| Nauplii |  |  |
| *Podon* spp. | Cladocera |  |
| *Bosmina* spp. |  |  |
| *Bosmina coregoni* |  |  |
| *Evadne nordmannii* |  |  |
| Calanoid Copepoda egg | other pelagic prey |  |
| Fish egg |  |  |
| Cypris larvae |  |  |
| Zoea larvae |  |  |
| Bivalvia | other intermediate prey | intermediate |
| Mysida |  |  |
| Megalopa larvae |  |  |
| *Diastylis rathkei* | *Diastylis rathkei* | benthic |
| Annelida | Annelida |  |
| Polychaeta |  |  |
| Teleostei | Teleostei |  |
| *Pomatoschistus minutus* |  |  |
| Halacaroidea | other benthic prey |  |
| Harpacticoid copepod |  |  |
| Gastropoda |  |  |
| *Hydrobia* spp. |  |  |
| Foraminifera |  |  |
| Amphipoda |  |  |
| Gammaridae |  |  |
| Ostracoda |  |  |
| *Crangon crangon* |  |  |

**SI4. Stomach content weights**

**Supporting Information Table SI4.1.** Number (N) of stomach samples of WBC per 10 mm-length class (numbers in brackets denote number of stomachs classified as empty), mean stomach content weight (SCW) (number in brackets displays standard deviation) and mean fullness index (number in brackets displays standard deviation).

| **Length class** | **N** | **Mean SCW** | **Mean Fullness** |
| --- | --- | --- | --- |
| 11-20 mm | 4(2) | 0.5 mg (± 0.0 mg) | 1.64 % (± 0.11%) |
| 21-30 mm | 17(0) | 0.7 mg (± 0.4 g) | 0.93 % (± 0.25 %) |
| 31-40 mm | 6(0) | 3.3 mg (± 3.6 mg) | 1.15 % (± 1.02 %) |
| 41-50 mm | 16(0) | 13.9 mg (± 8.5 mg) | 1.91 % (± 1.30 %) |
| 51-60 mm | 47(2) | 16.9 mg (± 10.6 mg) | 1.44 % (± 0.91 %) |
| 61-70 mm | 27(0) | 32.6 mg (± 26.0 mg) | 1.57 % (± 1.27 %) |
| 71-80 mm | 34(0) | 46.6 mg (± 42.4 mg) | 1.39 % (± 1.13 %) |
| 81-90 mm | 23(2) | 97.4 mg (± 73.0 mg) | 2.09 % (± 1.59 %) |
| 91-100 mm | 8(1) | 377.9 mg (± 258.0 mg) | 5.02 % (± 3.39 %) |
| 101-110 mm | 1(0) | 572.0 mg | 5.78 % |


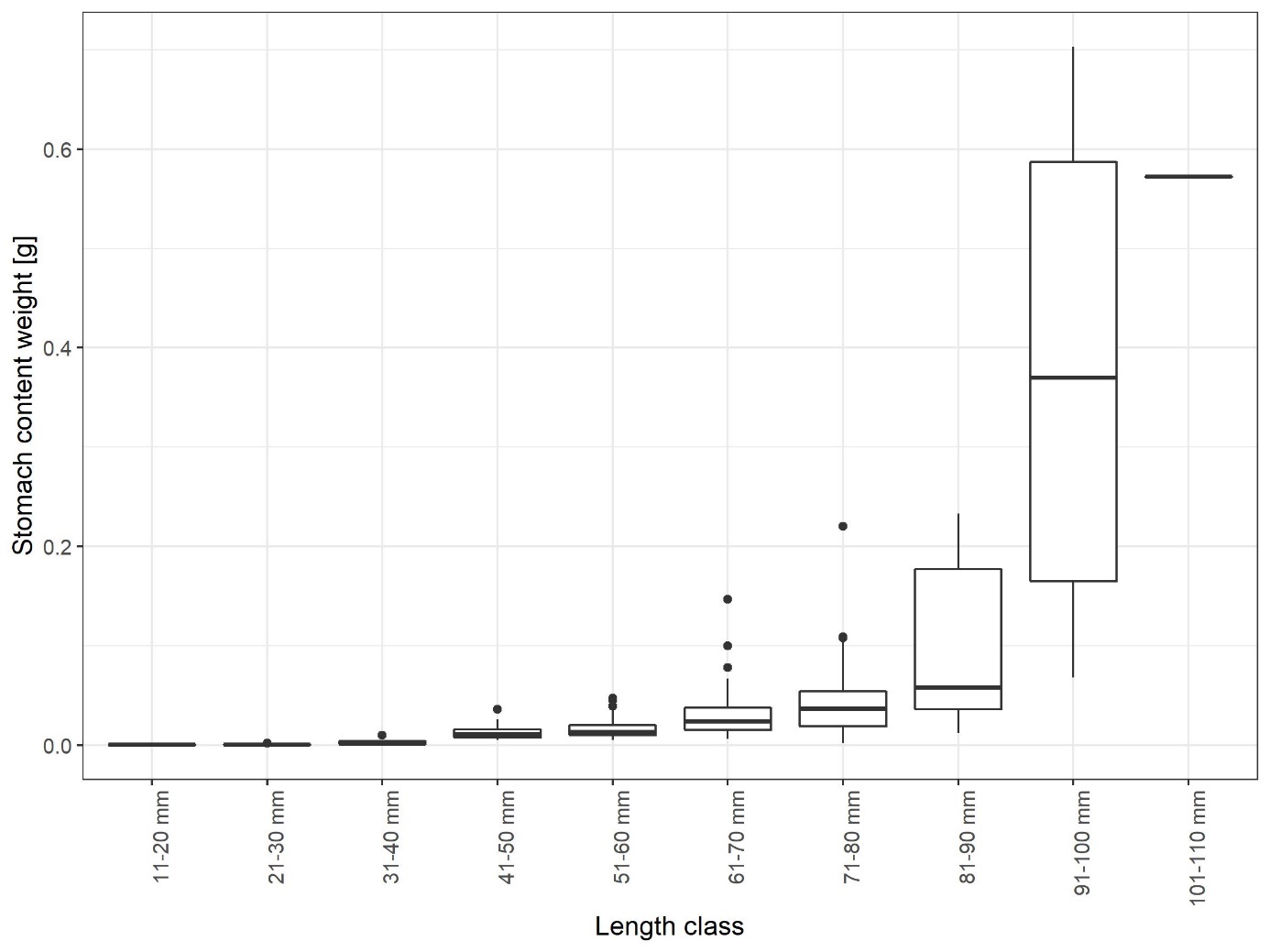


**Supporting Information Figure SI4.1.** Observed stomach content weights (SCW) of cod *Gadus morhua* from SD 22 per 10 mm length class. Boxplots show medians with first and third quartiles (hinges) of the observed SCW. Whiskers range from the upper/lower hinge to the largest value, but no further than 1.5 × IQR (interquartile range) from the hinge, respectively. Black dots represent outliers that are SCW above 1.5 × IQR from the upper hinge.

**SI5. Copepod prey composition per length class and sampling year**

**
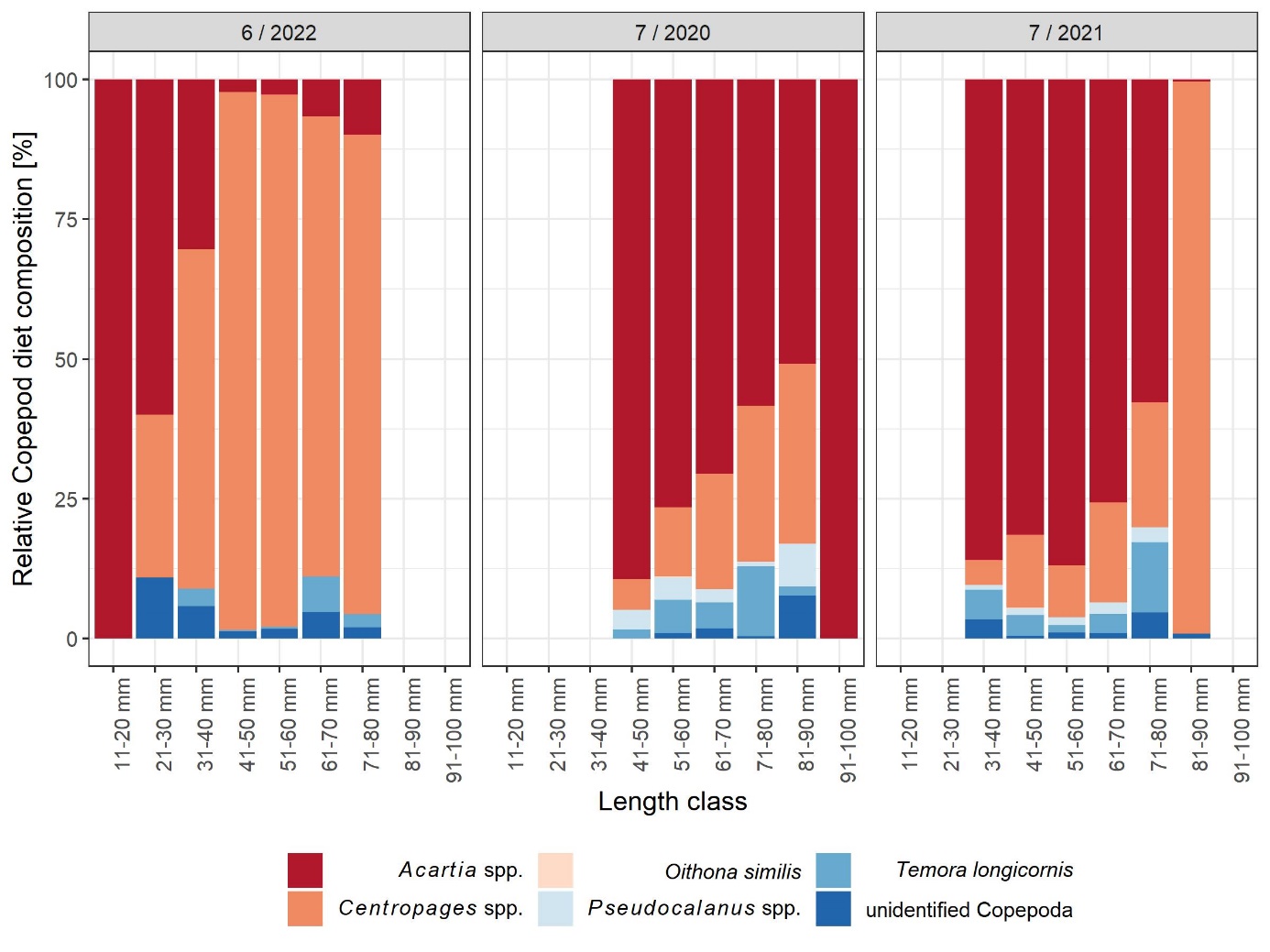
**

**Supplementary Figure SI5.1.** Mean relative Copepoda stomach contentcomposition (by recalculated prey wet weights) of juvenile cod *Gadus morhua* from SD 22 according to 10 mm length class and sampling date.

**SI6. Standard length to total length conversion of juvenile cod**

To be able to compare diet of 0-group cod with other studies (e.g., from Hüssy et al., 1997; Lomond, 1998) a total length-standard length relationship was created (R^2^ = 0.954) to enable a conversion of collected and calculated standard lengths (e.g. calculated L50 values) to total lengths. The conversion equation was based on 0-group cod (N = 794) from Stollergrund sampled in 2016 (Höper, 2016 unpublished).

$SL \left[ mm \right]=0.606015+0.904313*TL [mm]$ (Equation SI6)

with SL = standard length und TL = total length.

**References Supporting Information**

Costa, F.O., and Costa, M.H. (1999). Life history of the amphipod *Gammarus locusta* in the Sado estuary (Portugal). *Acta oecologica*, 20(4), 305-314.

Dawirs, R. R. (1980). Elemental composition (C, N, H) in larval and crab-1 stages of *Pagurus bernhardus* (Decapoda, Paguridae) and *Carcinus maenas* (Decapoda, Portunidae). *Marine Biology*, 57, 17-23.

Hansson, S., Rudstam, L. G., & Johansson, S. (1990). Are marine planktonic invertebrates food limited? The case of *Mysis mixta* (Crustacea, Mysidacea) in the Baltic Sea. *Oecologia*, **84**(3), 430-432.

Härkönen, T. (1986). *Guide to the otoliths of the bony fishes of the Northeast Atlantic*. Danbiu ApS. biological consultants, Hellerup, Denmark. 256pp.

Hernroth, L. (1985). Recommendations on methods for marine biological studies in the Baltic Sea, Baltic Marine Biologists, No. 10. *Institute of Marine Research, Lysekil*.

Höper, A. (2016). *Investigation and analysis of juvenile cod samplings.* [Unpublished laboratory analysis]. Institute for Marine Ecosystem and Fishery Science, University of Hamburg.

Hüssy, K., St. John, M.A., and Böttcher, U. (1997). Food resource utilization by juvenile Baltic cod *Gadus morhua*: a mechanism potentially influencing recruitment success at the demersal juvenile stage?. *Marine Ecology Progress Series, 155:* 199-208.

Jansson, A., Norkko, J., Dupont, S., and Norkko, A. (2015). Growth and survival in a changing environment: Combined effects of moderate hypoxia and low pH on juvenile bivalve *Macoma balthica*. *Journal of Sea Research*, 102, 41-47.

Lomond, T.M. (1998). Transition from pelagic to benthic prey for age group 0-1 Atlantic cod. *Fish. Bull*, *96*, 908-911.

Martel, A.L., Tremblay, R., Toupoint, N., Olivier, F., Myrand, B. (2014). Veliger size at metamorphosis and temporal variability in prodissoconch II morphometry in the blue mussel (Mytilus edulis): potential impact on recruitment. *Journal of Shellfish Research*, 33(2), 443-455.

Mohamedeen, H., Hartnoll, R.G. (1989). Larval and postlarval growth of individually reared specimens of the common shore crab *Carcinus maenas* (L.). *Journal of Experimental Marine Biology and Ecology*, 134(1), 1-24.

Rachor, E., Arntz, W. E., Rumohr, H., and Mantau, K. H. (1982). Seasonal and long-term population fluctuations in Diastylis rathkei (Crustacea: Cumacea) of Kiel Bay and German Bight. *Netherlands Journal of Sea Research*, 16, 141-150.

Rudstam, L.G. (1989). A bioenergetic model for Mysis growth and consumption applied to a Baltic population of *Mysis mixta*. *Journal of Plankton Research*, 11(5), 971-983.

Rumohr, H., Brey, T., and Ankar, S. (1987). A compilation of biometric conversion factors for benthic invertebrates of the Baltic Sea. *Institut für Meereskunde*.

Robinson, L.A., Greenstreet, S.P.R., Reiss, H., Callaway, R., Craeymeersch, J., De Boois, I., Degraer, S., Ehrich S., Fraser H.M., Goffin A., Kröncke I., Lindal Jorgenson L., Robertson M.R., Lancaster J. (2010). Length-weight relationships of 216 North Sea benthic invertebrates and fish. *Journal of the Marine Biological Association of the United Kingdom*, 90(1), 95-104.

Takahashi, K., Allan, W.H. (1984). Planktonic foraminifera: factors controlling sinking speeds. *Deep Sea Research Part A. Oceanographic Research Papers*, 31(12), 1477- 1500.
